# In-Vitro Anticancer and Antioxidant Activities of *Eremina desertorum* (Forsskal, 1775) Snail Mucin

**DOI:** 10.31557/APJCP.2021.22.11.3467

**Published:** 2021-11

**Authors:** Shimaa Attia Atta, Amina Mohamed Ibrahim, Fayed Attia Koutb Megahed

**Affiliations:** 1 *Department of Immunology, Theodor Bilharz Research Institute, Cairo, Egypt. *; 2 *Department of Environmental Research and Medical Malacology, Theodor Bilharz Research Institute, Giza, Egypt. *; 3 *Department of Nucleic Acid Research, Genetic Engineering and Biotechnology Research Institute, City of Scientific Research and Technological Applications, Alexandria, Egypt. *

**Keywords:** Eremina desertorum, mucin, antioxidant, anti-tumor, CACO-2, HepG-2

## Abstract

**Objectives:**

The aim of the present research is to elucidate the anti-oxidant and anti-tumor activities of the mucin extracted from Ereminia desertorum snails´ mucus against two types of tumor cell lines; human colon adenocarcinoma (CACO-2) cells and human hepatoma (HepG-2) cells.

**Methods:**

Both cell lines were treated with Ereminia desertorum snails´ mucin and the oxidative markers were measured in culture media and cells by biochemical and gene expression analysis using RT-PCR. The tumor suppressor gene expression was also evaluated using RT-PCR.

**Results:**

The culture media of HepG-2 or CACO-2 cells treated with the extract have high significant increased levels of catalase, SOD, GSH and total antioxidants. Apart from SOD in CACO-2 cells that didn’t differ from untreated cells. Also, Gene expression levels (2^^-ddct^) of the antioxidant markers in HepG-2 cells; GSTA-1, catalase, SOD, and GPx increased in mucin- treated cells. Also, these antioxidant genetic markers were up-regulated in CACO-2 cells by treatment with mucin extract. Gene expression levels (2^^-ddct^) of tumor suppression genes (p53, Rb, APC, and PTEN) in both HepG-2 and CaCO-2 cells were increased in mucin extract-treated cells.

**Conclusion:**

The present study highlighted the anti-oxidant and the anti-cancer activities of the mucin extracted from E. desertorum snails´ mucus that could attract attention to such natural product as a possible source of therapeutic compounds against liver and colon cancers.

## Introduction

Mollusca is the second largest invertebrate phylum (Sao Mai, 2014; Vinarski et al., 2020) that have a global distribution (Abd-El Azeem et al., 2020). They inhabited freshwater, marine and terrestrial habitat (Neubauer et al., 2015). Members of Mollusca have bioactive compounds that have anti-tumor, antibacterial, and antiviral activities (Rajaganapathy et al., 2000). 

Terrestrial snails are widely used in traditional medicine and as food resources because of their high content of protein (Ulagesan and Kim, 2018). These snails secrete mucus secretions called slime (Etim et al., 2016). It serves in protection of the animal from desiccation and infection by microorganisms due to its antimicrobial properties (Nantarat et al., 2019). This mucus consists of a mixture of mucopolysaccharides and glycoproteins (Skingsley et al., 2000), which may contribute to its beneficial pharmacological activities (Gabriel et al., 2011; Abd-El Azeem et al., 2020). 

Recently, there is a great interest about using snail mucus and its derivatives like mucin in wound healing, the treatment of skin disorders and muco-adhesive formulations (Hatuikulipi et al., 2016; Ali et al., 2018; Trapella et al., 2018). The slime of Achatina fulica snails could heal wound twice faster than the normal saline solution could do due to its content of glycosaminoglycan (Harti et al., 2016). Helix aspersa slime could ameliorate the experimentally induced colitis and reasoned these activities to its anti-inflammatory and antioxidant properties (Hatuikulipi et al., 2016). Another study on the slime of Helix aspersa maxima snail proved its anti-tumoral activity against human melanoma cells (Ellijimi et al., 2018). Also, the mucus from Helix aspersa muller has antimicrobial activity and several therapeutic properties, such as skin protection and wound repair due to its content of Helixcomplex; a mucopolysaccaride that contains small amounts of glycolic acid and allantoin (Trapella 2018; Gentili et al., 2020). Other land snails Achatina achatina and Achatina fulica crude mucus extracts have antimicrobial activity (Nantarat et al., 2019). 

The desert snails Eremina desertorum (Forsskal, 1775) (Gastropoda: Helicidae) live in sandy deserts and feeds on shrubs (Gabriel et al., 2011; Ali et al., 2016; Ali, 2017). Its mucus is used in wounds, superficial healing and muco adhesive formulations (Adikwu and Okafor, 2012; Hatuikulipi et al., 2016). 

Oxygen is a highly reactive atom that can be a part of damaging free radical molecules that possess unpaired electron. ROS reactive oxygen species (ROS) is a term which includes all reactive, oxygen-containing molecules (Cheeseman and Slater, 1993). Increased ROS production beyond the enzymatic and non-enzymatic antioxidant systems protective role results in oxidative stress which is a recognized cause of cancer development (Yoshikawa and Naito, 2002). Cancer development passes through three stages: Initiation, promotion, and progression. ROS is involved in all these stages via induction of nucleic acids, proteins, and lipids damage (Katakwar et al., 2016). Total oxidant status (TOS) and total antioxidant status (TAS) are parameters used to measure the general oxidative and antioxidant status of the body, respectively (Wu et al., 2017).

Hepatocellular carcinoma (HCC) is a malignant tumor that originates from hepatocytes and belongs to primary malignant tumors of the liver (Davis et al., 2008). It is the most prevalent malignancy of the liver and stages as the fifth most frequent cancer in men worldwide, and the seventh most dominant in women (Rashed et al., 2020). Colorectal cancer is ranked the fourth most common type of cancer (Rawla et al., 2019). It represents 13% of all malignant tumors in the gastrointestinal tract. It is the second most common cause of cancer-related death in males and females worldwide. Although there are multiple options available for treating cancers including curative resection, transarterial Chemoembolization, and radioembolization, the recurrence rate of the disease is still high and remains a huge problem (Kumari et al., 2018). Therefore, there is a necessity to investigate and evaluate novel chemo preventive strategies to lower the occurrence of the disease.

The aim of the present study is to investigate the anti-oxidant and anti-tumor activities of the mucin extracted from Ereminia desertorum snails´ mucus against two types of tumor cell lines; CACO-2 and HepG-2 cells. 

## Materials and Methods

*Handling of Eremina desertorum snails, slime and mucin*

*Animals*

The desert snails Eremina desertorum (Forsskal, 1775) (Gastropoda: Helicidae) were reared in medical malacology lab, Theodor Bilharz research institute. All individuals were cleaned with dechlorinated water to remove excretions on their mantles and shells. Snails were kept in plastic boxes (16 x 11x6 cm), temp., 26- 28 ^o^C and kept under high soil moisture (80% relative humidity) and fed on fresh leaves of green lettuce. 

*Snail slime and mucin extraction*

To have a pure fresh slime samples of the cleaned foot epithelium, snails were washed thoroughly and stimulated by rubbing a sterile wooden rod on its muscular foot, this force the snail to secret more slime; the slime was collected and kept in a sterile container and preserved at (- 30 °C) until use. 

The slime was macerated in water at 40°C for 24 hours. Fraction containing water-soluble slime was obtained from the procedure of mixing the water twice of the number of samples added to the slime. The supernatant was collected as WSF (Water Soluble Fraction). The slime fraction (mucin fraction) of the WSF was gained using ethanol precipitation technique by mixing the supernatant resulted from water maceration with absolute ethanol in a ratio of 1: 3, and centrifugation at 2900 r.p.m., for 30 minutes. The precipitation was re-dissolved with Tris -Cl and finally mucin fraction was obtained (Harti et al., 2016).

*Preparation of extract*

Ereminia desertorum snails´ mucin was warmed to 37°C and filtered using syringe filter (45µm pore size). The sterile mucin was collected in sterile tube and used immediately.

*Cell culture*

*Cell line revival and propagation*

HepG-2 and CACO-2 cell lines were obtained from Immunology lab, Theodor Bilharz Research institute. Cells were revived and cultured in RPMI (Gibco) supplemented with 1% penicillin/streptomycin (Sigma Aldrich), 1% L glutamine (Sigma Aldrich) and 10% Fetal bovine serum (Gibco). Tissue culture flasks containing the cells were incubated in 5% CO_2_ humidified incubator (Thermo) till reaching confluency.

*Applying the mucin to cell lines*

On full confluency, media were discarded and replaced with serum free RPMI supplemented with 0.2µl/ml of the Ereminia desertorum snails´ mucin. Negative control (cells cultured in RPMI only) and positive control (cells cultured in RPMI and lead nitrate 50mM/ml) were included. Flasks were incubated overnight in 5% CO_2_ incubator.

*Cell trypsinization*

Media were collected for measuring the anti-oxidant markers and flasks were washed with PBS (Gibco) and cells were trypsinized by 0.25% Trypsin-EDTA solution (Sigma Aldrich). Flasks were incubated at 37°C for 2 min. Trypsin was deactivated by adding equal volume of culture media and cells were collected, washed with PBS twice by centrifugation and subjected to gene analysis by RT-PCR.

*Gene expression analysis*

*RNA extraction*

RNA was isolated using the RNeasy Kit (Qiagen, Chatsworth, CA) according to the manufacturer’s instructions. One microgram of cellular RNA was reverse transcribed into cDNA using SuperScript II reverse transcriptase and random hexamer primers (Invitrogen Life Technologies, USA). 

*RT-PCR*

The PCR reaction was carried out in a volume of 10 µl in a mixture that contained appropriate sense- and anti-sense primers and a probe in TaqMan Universal PCR Master Mixture (Applied Biosystems, Foster City, California). We used the Assays-on-DemandTM Gene Expression products, which consist of a 20x mix of unlabeled PCR primers and TaqMan MGB probe (FAMTM dye-labeled). These assays are designed for the detection and quantification of specific human genetic sequences in RNA samples converted to cDNA. The primers used are illustrated in Table 1. Real-time PCR amplification and data analysis were performed using the A7500 Fast Real-Time PCR System (Applied Biosystems). Each sample was assayed in duplicate in a MicroAmp optical 96-well plate. The thermo-cycling condition consisted of 2 minutes at 50°C and 10 min incubations at 95°C, followed by 40 two-temperature cycles of 15 seconds at 95°C and 1 min at 60°C. Gene expression was normalized to internal controls and fold changes were calculated using the relative quantification method (2^−ΔΔCq^).

*Statistical analysis*

One Way ANOVA was used to test the effect of the extract on the measured parameters of tested cells in comparison to the other two groups.

## Results

*Oxidative markers*

**Figure 1:**
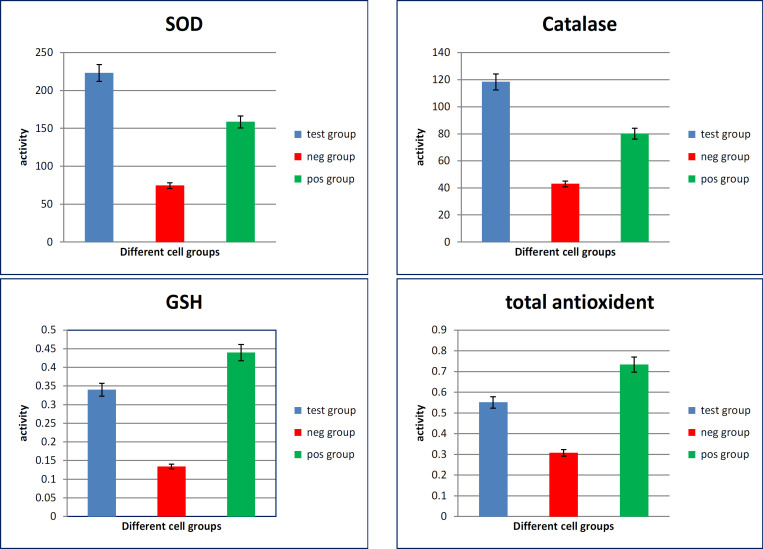
Oxidative Stress Markers Measured in Culture Media of Different HepG-2 Cell GAroups. SOD, superoxide dismutase; GSH, reduced glutathione

The antioxidative markers in mucin extract treated-culture media of HepG-2 cells showed high significant increase in the activity of catalase, SOD, GSH and total antioxidants as compared with their levels in media of untreated cells (p value <0.001) (Figure 1). 

**Figure 2:**
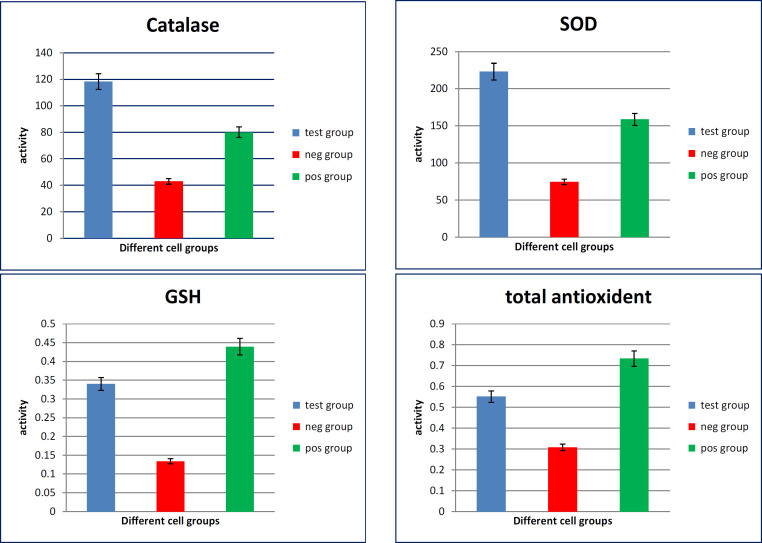
Oxidative Stress Markers Measured in Culture Media of Different CACO_2_ Cell Groups. SOD, superoxide dismutase; GSH, reduced glutathione

The antioxidative markers in mucin extract treated-culture media of CACO-2 cells showed high significant increase in the activity of catalase, SOD, GSH and total antioxidants as compared with media of untreated cells (p value <0.001). While SOD didn’t show a significant difference when compared to the untreated cells (Figure 2). 

*Gene expression analysis*

*Antioxidant genetic markers*

**Figure 3:**
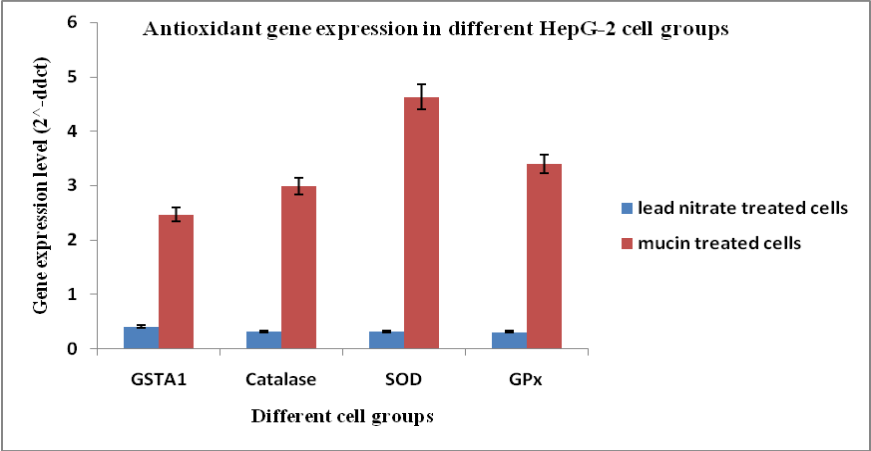
Antioxidant Gene Expression Levels in Different HepG-2 Cell Groups. SOD, Superoxide dismutase; GPx, Glutathione peroxidase; GSTA1, Glutathione S-transferase A1

Gene expression levels (2^^-ddct^) of Antioxidant markers in HepG-2 cells; GSTA-1, catalase, SOD, and GPx showed increased expression in cells treated with the mucin extract by (2.46, 2.98, 4.62 and 3.40), respectively when compared to the cells treated with lead nitrate (0.40, 0.32, 0.32 and 0.31). Gene expression levels in untreated control cell group equal (1) in data analysis and calculation. That means that the antioxidant genetic markers were up-regulated in HepG-2 cells by treatment with mucin extract and controversial, down-regulated by treatment with lead nitrate (Figure 3).

**Figure 4:**
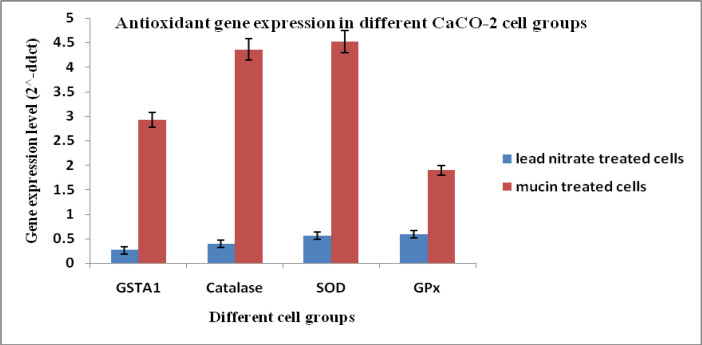
Antioxidant Gene Expression Levels in Different CaCO_2_ Cell Groups. SOD, Superoxide dismutase; GPx, Glutathione peroxidase; GSTA1, Glutathione S-transferase A1

On the other hand, gene expression levels (2^^-ddct^) of Antioxidant markers in CaCO-2 cells; GSTA-1, catalase, SOD, and GPx showed increased expression in cells treated with the mucin extract by (2.92, 4.35, 4.51 and 1.89), respectively when compared to the cells treated with lead nitrate (0.26, 0.39, 0.56 and 0.59). Gene expression levels in untreated control cell group equal (1) in data analysis and calculation. That means that the antioxidant genetic markers were up-regulated in CaCO-2 cells by treatment with mucin extract and controversial, down-regulated by treatment with lead nitrate (Figure 4). 

*Tumor suppression genes analysis*

**Figure 5:**
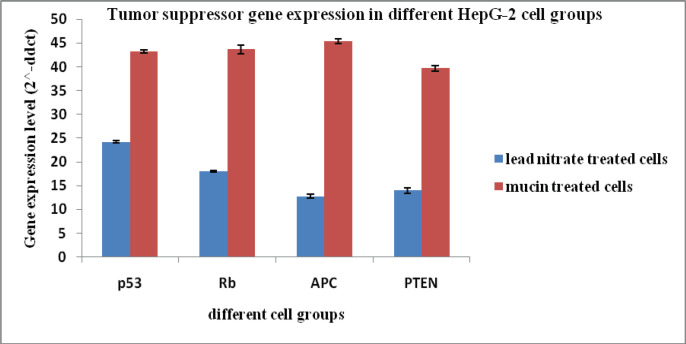
Tumor Suppression Genes Levels in Different HepG-2 Cell Groups. p53, Tumor protein p53; Rb, retinoblastoma gene; APC, Adenomatous polyposis coli; PTEN, Phosphatase and tensin homolog protein

Gene expression levels (2^^-ddct^) of tumor suppression genes in HepG-2 cells; p53, Rb, APC, and PTEN showed increased expression in cells treated with the mucin extract by (43.2, 43.6, 45.4 and 39.6), respectively when compared to the cells treated with lead nitrate (24.2, 17.9, 12.8 and 13.9). Gene expression levels in untreated control cell group equal (1) in data analysis and calculation (Figure 5).

**Figure 6:**
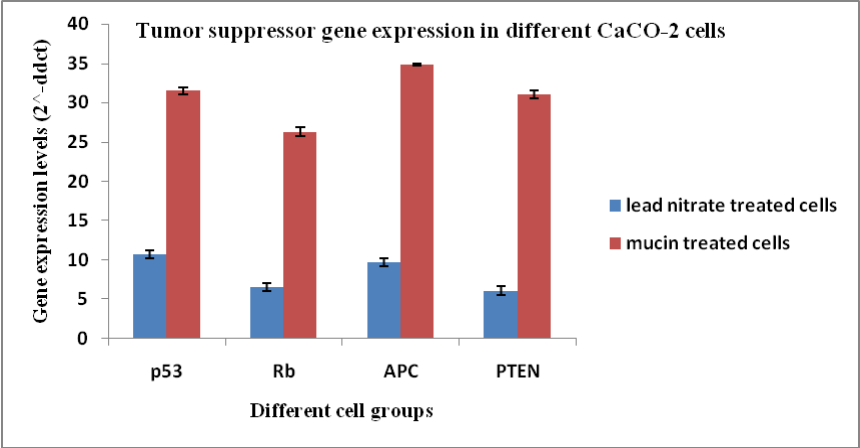
Tumor Suppression Genes Levels in Different CACO_2_ Cell Groups. p53, Tumor protein p53; Rb, retinoblastoma gene; APC, Adenomatous polyposis coli; PTEN, Phosphatase and tensin homolog protein

On the other hand, gene expression levels (2^^-ddct^) of tumor suppression genes in CaCO_2_ cells; p53, Rb, APC, and PTEN showed increased expression in cells treated with the mucin extract by (31.5, 26.2, 34.8 and 31.0), respectively when compared to the cells treated with lead nitrate (10.7, 6.5, 9.6 and 6.0). Gene expression levels in untreated control cell group equal (1) in data analysis and calculation (Figure 6). 

**Table 1:** Primers Sequences for RT-PCR

Symbol	Gene	Primer sequence	Reference
P53	Tumor protein p53	F: 5’-ATGTTTTGCCAACTGGCCAAG-3’	Mitupatum et al. (2016)
		R: 5’-TGAGCAGCGCTCATGGTG-3’	
Rb	retinoblastoma gene	F: 5'-ATGCCCCAGAACCCTTGTATC-3'	Wu et al. (2017)
		R: 5'-GCCCATAGCCTTCCTTCTGAT-3’	
APC	Adenomatous polyposis coli	F: 5’- ACCTCATCATTACACGCCTATT -3’	Zhang et al. (2015)
		R: 5’- CCTTATTTTCTTTTGCCTTTCT-3’	
PTEN	Phosphatase and tensin homolog protein	5’- ACAGGC-TCCCAGACATGACA-3’	Ma et al. (2005)
		5’- TCAG-ACTTTTGTAATTTGTGTATG-3’	
GSTA1	Glutathione S-Transferase Alpha 1	F: 5’-AGCCGGGCTGACATTCATCT-3’	Razali et al. (2010)
		R: 5’-TGGCCTCCATGACTGCGTTA-3’	
CAT	Catalase	F: 5′-CCAGATCAGTAGCGTGGCACG-3′	Kampkötter et al. (2007)
		R:5′-CATAGCGTGCGGTTTGCTGTGC-3′	
SOD	Super oxide dismutase	F: 5’-ACTGGTGGTCCATGAAAAAGC-3’	Zhang et al. (2015)
		R: 5’-AACGACTTCCAGCGTTTCCT-3’	
GPx	Glutathione peroxidase	F: 5’- CACAACGGTGCGGGACTA -3’	Chen et al. (2016)
		R: 5’- CATTGCGACACACTGGAGAC -3’	
β-actin	Beta-actin	F: 5-TCCCTGGAGAAGAGCTACG-3’	Sharula and Zhongjun. (2017)
	(reference gene)	R: 5-GTAGTTTCGTGGATGCCACA-3’	

## Discussion

Terrestrial snail mucus consists of a mixture of mucopolysaccharides and glycoproteins that have many pharmacological activities (Abd-El Azeem et al., 2020). These bioactive components have cytotoxic, anti-inflammatory, antimicrobial, and anti-tumor activities (Anand and Edward, 2002). They are also involved in variety skin care compositions as they have anti-aging properties (Dolashka et al., 2015). 

Oxidative stress can be involved in the development of chronic and degenerative illness such as cancer, neurodegenerative diseases, autoimmune disorders, and rheumatoid arthritis (Pham-Huy et al., 2008). Reactive oxygen species (ROS) are the main cause of the oxidative stress in the body (Rezaie et al., 2007). To maintain the balance in the body, the endogenous antioxidant system scavenged ROS and the free radicals (Halliwell, 2006). The endogenous anti oxidative enzymes such as superoxide dismutase (SOD), catalase (CAT) and glutathione peroxidase (GPx) are able to protect the liver against oxidative damage and SOD and SOD catalyzes the dismutation of the superoxide anion radical (O2) to oxygen and hydrogen peroxide (H_2_O_2_). H_2_O_2_ may then be converted into water by CAT. By contrast, GPx is able to protect the cell against oxidative stress via the reduction of H_2_O_2_ and lipid peroxides, using glutathione as an electron donor (Kumar et al., 2012). Glutathione S-transferases (GSTs) are involved in the detoxification of xenobiotics such as toxins and carcinogens (Eaton and Bammler, 1999).

The main function of the antioxidants is depending on its reducing power of the oxidative damage (Bashir et al., 2015). Many antioxidants compounds are present in natural product such as snail hemolymph (Shittu et al., 2015), and mucus (Gabriel et al., 2011).

The present results revealed that the culture media of HepG-2 cells treated with the extract have high significant increased levels of catalase, SOD, GSH and total antioxidants. Also, there were high significant increased levels of catalase, GSH and total antioxidants in culture media of CACO_2_ cells treated with extract. While SOD level didn’t show significant difference between the treated and the untreated cells. Also, the present investigation confirmed that antioxidant genetic markers were up-regulated in HepG-2 cells by treatment with mucin extract. Where Gene expression levels (2^^-ddct^) of the antioxidant markers; GSTA-1, catalase, SOD, and GPx showed increased expression in media of HepG2 cells treated with the mucin extract. Also, antioxidant genetic markers were up-regulated in CACO-2 cells by treatment with mucin extract.

These antioxidant properties were reported and confirmed in Cornu aspersum mucus due to the presence of allantoin that has antioxidant activities (Kostadinova et al., 2018). Also, Brieva et al., (2008) found that slime from Helix aspersa garden snail contains antioxidant superoxide dismutase and glutathione-s-transferase activity. Chen et al., (2019) stated that pretreatment with Ligustrum robustum polyphenol extract lead to cytoprotective effect against Hydrogen Peroxide-Induced Oxidative Stress, where it decreased ROS level, and maintaining the endogenous antioxidant system through increasing of SOD, CAT, GSH-Px, and GR activities compared to the H_2_O_2_ group. It could be attributed that the high dosage extracts may exert antioxidant activities by directly scavenging free radicals and its reducing capacity. 

The main objective of treating cancer is to promote the death of cancer cells without causing damage to normal cells (Gerl and Vaux, 2005). P53 protein is one of the tumor suppressors that help in the removal of DNA-damaged cells and in arresting the cell cycle (Chiang et al., 2014). Another tumor suppressor is the retinoblastoma protein (RB) (Agarwal et al., 1998). It regulates the cell proliferation and suppresses cancer growth (Goodrich, 2006; Ebata et al., 2016). The adenomatous polyposis coli (APC) gene is a key tumor suppressor gene that is involved in cell adhesion and migration, organization of the actin and microtubule networks, spindle formation and chromosome segregation (Aghabozorgi et al., 2019). Also, phosphatase and tensin homolog deleted on chromosome ten (PTEN) is the key negative regulator of the phosphatidylinositol 3-kinase (PI3K) signaling pathway that promotes cell survival and proliferation and is frequently deregulated in various human cancers (Stambolic et al., 1998). Mutations of the PTEN gene arise during cancer progression (Ali et al., 1999).

The present results showed that gene expression levels of p53, Rb, APC, and PTEN were increased in both HepG-2 and CACO-2 cells treated with the mucin extract. These results are in good accordance with Teerasak et al., (2016) who confirmed the presence of two fractions of proteins in the African snail Achatina fulica mucus that can decrease the viability of breast cancer cells (MCF-7). Also, the mucus of Actinia equine showed cytotoxic effect on human erythromyeloid leukemia-derived cells (K592) (Stabili et al., 2015). El Ouar et al., (2017) showed that the extracts from tissues of Helix Aspersa Müller possessed anticancer activity against breast cancer cells (Hs578T).

Conclusively, the present study highlights the anti-oxidative and the anti-cancer activities of the mucin extracted from E. desertorum snails´ mucus against two types of tumor cells; CACO-2 and HepG2 cells. This could be helpful for the production of a natural cancer therapy.

## Author Contribution Statement

None.
